# Intracellular ‘wiring’ for real-time cell communication

**DOI:** 10.3402/nano.v4i0.22429

**Published:** 2013-08-13

**Authors:** F. J. Rawson, C. L. Yeung, S. K. Jackson, P. M. Mendes

**Affiliations:** 1 School of Chemical Engineering, University of Birmingham, Edgbaston, UK; 2 School of Biomedical and Biological Sciences, University of Plymouth, Portland Square, Drake Circus, Plymouth, Devon, UK

**Fig. 1:**
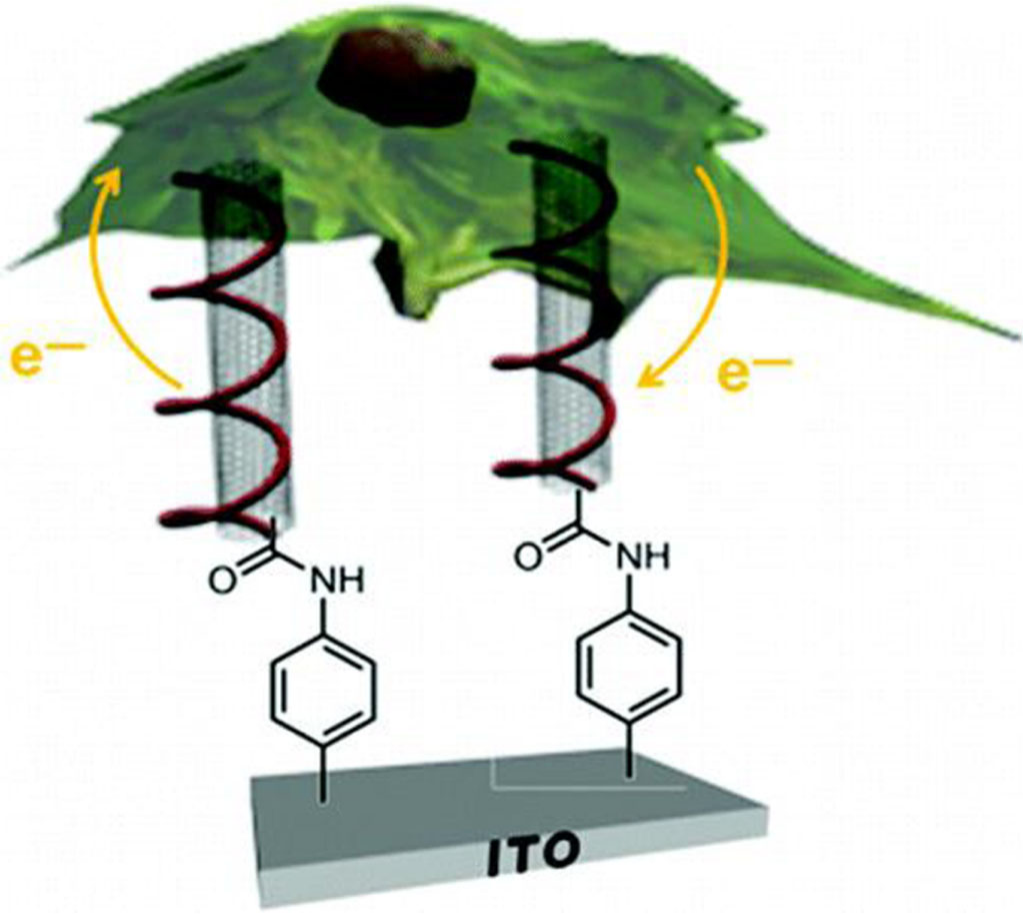
Diagrammatic representation showing CNTs capable of being taken up naturally by a mouse macrophage cell and subsequently to be used for intracellular electrochemical sensing of the redox active methylene blue moiety.

Technology for understanding the real-time molecular events occurring within cells that underpins their behaviour is currently lacking. Despite important developments, the biochemical processes in a cell can be only poorly quantified, limiting the ability to resolve the dynamic molecular processes that underlie important cell-fate decisions, such as differentiation, cell division and cell death. The regulation of cell fate lies at the core of most aspects of cell biology from normal development to malignancy. It is only by a detailed knowledge of how cells work, independently and together, in healthy and disease states that one will be capable of understanding and anticipating the onset and effects of disease, and therefore creating appropriate and effective means to prevent and treat disease. In the last few years, research groups across the world have begun to focus their efforts towards developing various types of nanosensors in addressing this grand challenge. The technology has the potential to monitor spatial and temporal changes of cellular chemistry, which can shed new light on cell's function, as they sense on an equivalent molecular scale. Electrochemical detection inside cells offers several advantages over the conventional fluorescence measurements. It includes less expensive components with simple sample preparation steps, and of utmost importance, the capability of measuring quantities down to the zeptomole level – allowing detection of trace levels of electroactive species – in complex turbid environments. Towards this aim, Dr. Rawson, Dr. Mendes, and coworkers (1) within the School Chemical Engineering at the University of Birmingham and collaborator Prof. Jackson at the University of Plymouth, recently reported in *Nano Letters* (2013; 13(1): p. 1–8) the first intracellular electrochemical (Fig. 1) sensing platform. The technology developed provides an alluring platform to monitor and determine the precise location of redox activities occurring within the cell.

Their electrochemical sensing platform was fabricated using indium tin oxide, which is the underlying conducting surface. The surface was modified with a molecular building block, acting as an anchor, allowing for the self-assembly of highly conductive vertically aligned carbon nanotubes (CNTs). Atomic force microscopy was used to show that the CNTs were approximately 60 nm in height, thus having dimensions sufficient enough to cross the plasma membrane. Self-assembled vertically aligned CNTs were further modified with DNA. The DNA modification allowed the CNTs to be naturally taken up by a macrophage cell (RAW 264.7). With CNTs having dimensions down to a few nanometres, these 3D nanostructured surfaces are unparalleled as high spatial resolution tools for cell biology. The team is now working on significantly improving their technology for intracellular sensing of reactive oxygen species. This will allow for a greater understanding as to their role in cell signalling in response to bacterial stimulation.
